# Maternal participant experience in a South African birth cohort study enrolling healthy pregnant women and their infants

**DOI:** 10.1186/s13010-016-0036-2

**Published:** 2016-07-19

**Authors:** Whitney Barnett, Kirsty Brittain, Katherine Sorsdahl, Heather J. Zar, Dan J. Stein

**Affiliations:** Department of Paediatrics and Child Health, Red Cross War Memorial Children’s Hospital, and Medical Research Council Unit on Child & Adolescent Health, University of Cape Town, Cape Town, South Africa; Alan J. Flisher Centre for Public Mental Health, Department of Psychiatry, University of Cape Town, Cape Town, South Africa; Department of Psychiatry and Mental Health, University of Cape Town, Cape Town, South Africa; Medical Research Council Unit on Anxiety & Stress Disorders, Cape Town, South Africa

**Keywords:** Participant experience, Research burden, Respondent satisfaction, Healthy volunteer

## Abstract

**Background:**

Critical to conducting high quality research is the ability to attract and retain participants, especially for longitudinal studies. Understanding participant experiences and motivators or barriers to participating in clinical research is crucial. There are limited data on healthy participant experiences in longitudinal research, particularly in low- and middle-income countries. This study aims to investigate quantitatively participant experiences in a South African birth cohort study.

**Methods:**

Maternal participant experience was evaluated by a self-administered survey in the Drakenstein Child Health Study, a longitudinal birth cohort study investigating the early life determinants of child health. Pregnant mothers, enrolled during the second trimester, were followed through childbirth and the early childhood years. Satisfaction scores were derived from the participant experience survey and quantitatively analyzed; associations between satisfaction scores and sociodemographic variables were then investigated using a linear regression model.

**Results:**

Data were included from 585 pregnant mothers (median age 26.6 years), who had participated in the study for a median time of 16 months. Overall participant satisfaction was high (median score 51/60) and associated with increased attendance of study visits. Reasons for participating were a belief that involvement would improve their health, their child’s health or the health of family and friends. Potential reasons for leaving the study were inconvenience, not receiving clinical or study results, and unexpected changes in study visits or procedures. Variables associated with higher overall satisfaction scores were no prior participation in research, higher socioeconomic status, less intensive follow-up schedules and having experienced stressful life events in the past year.

**Conclusions:**

Satisfaction scores were high and associated with increased visit attendance. Participants’ perceived benefits of study participation, most notably the potential for an improvement in the health of their child, were a significant motivator to enroll and remain in the study. The consistent theme of perceived health benefits as a motivator to join and remain in the study raises the question of whether participation in research results in actual improvements in health.

## Background

The ability to attract and retain participants is critical to conducting high quality research. This is particularly important for longitudinal studies, since high cohort retention over long periods of time is required. Successful enrolment and retention is often predicated on appropriate consenting, well-trained staff, effective communication with participants and a favorable risk/benefit balance. Researchers ensure that these aspects are met through process compliance to ensure participant safety, by assessing staff competency, by meeting ethical and regulatory requirements and through community engagement [1]. Typically, this does not involve feedback from study participants or ongoing evaluation of their experience, motivations or barriers to participation. In recent years researchers have begun to address this gap with the goal of meeting enrolment targets, improving retention and to provide operational feedback regarding participant experiences [2–6].

Most studies investigating research participant experience have focused on randomized clinical trials (RCTs) [2, 7–19]. Motivators for RCT enrolment included access to specialized treatment, perceived personal benefit, [7–10, 14, 17, 18] contribution to science, [10] the ability to imbue their experience with value [9] and altruism [2, 11, 12]. Some studies have found altruism to be a primary motivator, [2, 11, 12] though others have found it to be a conditional altruism balanced against participant’s perceived personal benefits [5, 7–9, 12–15]. Given that enrolment in a RCT typically offers participants the potential for treatment or specialized medical care; the motivation and experience of participating in other types of studies are likely to yield different findings. However, there are limited data on participant experiences in observational studies involving lengthy follow up, as well as participant experiences in low and middle-income countries (LMICs). Further, most studies reporting participant experience have included participants living with a specific disease, rather than healthy participants [5, 20, 21].

Given the lack of data on healthy participant experiences in longitudinal observational studies, particularly in LMIC settings, we investigated participant experience in a longitudinal African birth cohort study utilizing a self-administered survey. We aimed to: 1) describe healthy participant experiences; 2) identify factors associated with increased participant satisfaction; and 3) evaluate participant experiences, motivators and barriers to participation.

## Methods

### Study design

**Fig. 1:**
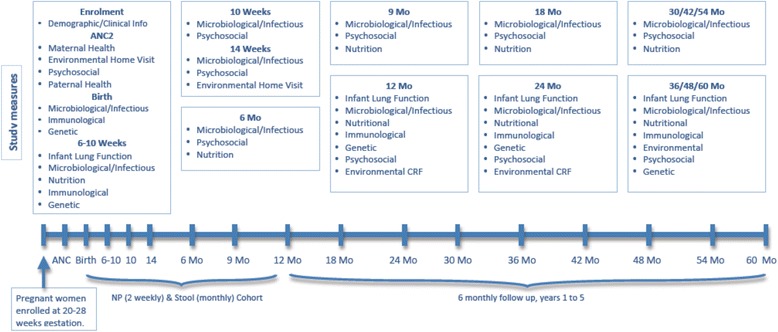
Study follow up visits

The Drakenstein Child Health Study (DCHS) is a multidisciplinary birth cohort study investigating the epidemiology and etiology of childhood respiratory illness and the determinants of child health in a peri-urban area in South Africa [22]. Mothers were enrolled during the second trimester of pregnancy and mother-infant pairs are followed until children reach at least 5 years of age. Mother-infant pairs attend numerous visits during this period; at enrolment mothers were able to choose to participate in usual study follow-up (main cohort) or an intensive cohort, in which 2 weekly follow-up with nasopharyngeal sampling was done in children throughout the first year of life (Fig. 1). Visits included questionnaires, clinical examination, specimen collection, lung function testing, psychosocial assessments, infant developmental measures and home visits.

### Setting

The DCHS is located in the Drakenstein subdistrict, a peri-urban area 60 km outside Cape Town, South Africa, with a population of approximately 200,000 [22]. More than 90 % of the population access health care in the public sector including antenatal and child health services [23]. Similar to many LMICs, the area has a high burden of childhood disease, including pneumonia, [24] and a high prevalence of risk factors for childhood illness, such as tobacco smoke exposure, drug use, interpersonal violence, overcrowding, malnutrition and poverty [25].

### Participants

Pregnant women were recruited from two primary health care clinics serving distinct populations – TC Newman clinic (serving a mixed race population) and Mbekweni clinic (serving a black African population). Enrolment commenced in March 2012. Exclusion criteria included women who were planning to move out of the area, women under 18 years of age, lack of informed consent or not attending study clinics for antenatal care. All mothers were asked to complete a participant experience survey at the 12 month postnatal study visit. Participants were provided with reimbursements to cover travel expenses related to study visit attendance.

### Participant experience survey

We evaluated participant experience using an adapted version of the Research Participant Experience Survey [26, 27]. Adaptations included adjusting questions to be relevant for healthy volunteers and local context and shortening the questionnaire for acceptability. The survey was self-administered in a private space, allowing for greater anonymity. Questionnaires were available in the first language (Afrikaans, English or Xhosa) of a participant; translations were completed by trained local translators to ensure that these were culturally appropriate.

The participant experience survey assessed reasons for joining and staying in the study, reasons participants may have considered leaving the study and participant satisfaction overall. Satisfaction was assessed across several themes, including satisfaction with study information, study staff, experience versus expectations, study procedures and overall experience; themes were constructed based on focus group research used to develop the Research Participant Experience Survey [27]. A scoring system was devised, where responses indicating greater levels of satisfaction with study experiences were scored higher than those indicating dissatisfaction. Satisfaction scores for each theme in the questionnaire were calculated by summing individual item responses relating to each respective theme; theme specific content is detailed below.

Satisfaction with study staff was assessed based on the quality of participant relationships with study staff, how closely study staff kept participants informed of study aspects and whether staff were accessible for questions (maximum = 6). Satisfaction with study procedures was assessed based on the amount of discomfort and duration of specimen collection and the acceptability of procedures such as lung function testing and psychosocial questionnaires and evaluations (maximum = 8). Satisfaction with study information was based on whether participants felt well informed of study procedures and well prepared for what they experienced (maximum = 6). Participants were asked about overall study experience, including whether they would participate in a similar study again, whether they would recommend participation to family or a friend and whether their overall experience was good or bad (maximum = 7). Finally, participants were asked about their experience versus expectation, specifically whether participation was better than they expected or more difficult than they expected (maximum = 2). Individual theme scores were then summed to create an overall satisfaction score (maximum = 60).

Sociodemographic characteristics, including measures of socioeconomic status, were assessed at enrolment. A composite SES score was developed based on employment status and standardized scores of educational level, household income and a composite asset index made up of access to household resources, amenities and market access. Participants were categorized as low SES, low-moderate SES, moderate-high SES or high SES. A validated questionnaire, the World Mental Health Life Events Questionnaire, was used to assess stressful life events for participants based on items included in the South African Stress & Health Study [28]. For this analysis, we used a threshold of having experienced three or more stressful life events in the previous 12 months.

### Data analysis

Differences in participant motivators and study experiences across recruitment site and cohort (main versus intensive cohort) were identified using χ^2^ or Fisher exact tests for categorical variables and Wilcoxon rank sum tests for continuous variables. The association between participant experience scores and the number of scheduled study visits attended was explored using Spearman’s rank correlation. Variables significantly associated with higher participant experience scores (at *p* < 0.05) were identified using Wilcoxon rank sum tests for dichotomous variables and Kruskal-Wallis one-way analysis of variance tests for categorical variables, and were included in a multivariate model of participant experience using a forward stepwise approach. A linear regression model was built using likelihood ratio tests to assess model fit. Data were analyzed using Stata 12 (StataCorp Inc, College Station, Texas, USA).

## Results

From March 2012 to March 2015, 1,225 mothers were enrolled with 1,139 births; 125 (10 %) of children were lost to follow up prior to reaching 12 months of age, the time point at which the participant experience survey was administered. Most (55 %) loss to follow up was due to participants moving out of the area or being unable to attend study visits. At the time data were analyzed, 627 children were still active in the study and had reached 12 months of age; of these, 585 participants had complete data and were included in the present analysis. At the time of survey administration, participants had been enrolled in the cohort for a median time of 16 months (IQR 15–17) and had been scheduled to attend between 6 and 29 study visits (the latter includes the bi-weekly study schedule for the intensive cohort, Fig. 1). Participants had a median age of 26.6 years (IQR 22.4–31.3) at enrolment, with a low proportion of stable relationships, low levels of employment and low socioeconomic status, Table 1. Only 18 % of participants had previously been involved in a research study, with participants from Mbekweni clinic significantly more likely to report previous involvement in research. The participant experience survey adapted for this study shows good internal consistency, with a Cronbach’s alpha coefficient of 0.83.

**Table 1:** Baseline sociodemographic characteristics

Variable	Mbekweni – *n* (%)	TC Newman – *n* (%)	Total sample – *n* (%)	*P*-value
Number of mothers	300 (51)	285 (49)	585 (100)	
Cohort				
Main	1 (0)	141 (49)	142 (24)	<0.001
Intensive	299 (100)	144 (51)	443 (76)	
Race				
Black	298 (99)	3 (1)	301 (51)	<0.001
Mixed race	2 (1)	282 (99)	284 (49)	
Median age at enrolment (IQR)	27.4 (22.6–32.1)	25.5 (22.1–30.1)	26.6 (22.4–31.3)	0.006
Marital status				
Married/cohabiting	104 (35)	117 (41)	221 (38)	0.111
Gravidity				
Primigravida	87 (29)	112 (39)	199 (34)	0.009
Highest level of education				
Some secondary	197 (66)	175 (61)	372 (64)	0.284
Completed secondary	103 (34)	110 (39)	213 (36)	
Current employment status				
Employed	58 (19)	80 (28)	138 (24)	0.013
Average household income				
< R1000/month	153 (51)	107 (38)	260 (44)	
R1000-R5000/month	120 (40)	133 (47)	253 (43)	0.002
> R5000/month	27 (9)	45 (16)	72 (12)	
Composite SES quartile				
Lowest SES	94 (31)	47 (16)	141 (24)	
Low-moderate SES	81 (27)	51 (18)	132 (23)	<0.001
Moderate-high SES	63 (21)	89 (31)	152 (26)	
High SES	62 (21)	98 (34)	160 (27)	
Prior participation in research	71 (24)	34 (12)	105 (18)	<0.001

### Overall satisfaction

Participant satisfaction was high overall, with a median score of 51 (IQR 46–56) out of a maximum score of 60. Many of the thematic satisfaction scores differed between clinic and between cohort (main versus intensive), Table 2. Mbekweni clinic participants had a significantly lower median satisfaction score of 47/60 (IQR 42–51) compared to participants at TC Newman [score 55/60 (IQR 50–58; *p* < 0.001)]. Intensive cohort participants also had a significantly lower median score of 50 (IQR 44–54) compared to that of main cohort participants, 54 (IQR 49–57; *p* < 0.001).

**Table 2:** Participant experiences and visit attendance

Participant experiences across site				
	Median score (IQR) across site			*P*-value
Variable	Mbekweni	TC Newman	Total sample	
Experiences with study information (maximum: 6)	5 (4–6)	6 (5–6)	6 (4–6)	<0.001
Experiences with study staff (maximum: 6)	6 (5–6)	6 (5–6)	6 (5–6)	0.633
Experiences with study procedures (maximum: 8)	2 (0–4)	7 (3–8)	3 (1–7)	<0.001
Experience vs expectations (maximum: 2)	1 (1–2)	1 (1–2)	1 (1–2)	0.615
Overall experiences (maximum: 7)	7 (6–7)	7 (6–7)	7 (6–7)	<0.005
Total participant experience score (maximum: 60)	47 (42–51)	55 (50–58)	51 (46–56)	<0.001
Participant experiences across cohort				
	Median score (IQR) across cohort			
Variable	Main cohort	Intensive cohort	Total sample	*P*-value
Experiences with study information (maximum: 6)	6 (5–6)	6 (4–6)	6 (4–6)	0.001
Experiences with study staff (maximum: 6)	6 (5–6)	6 (5–6)	6 (5–6)	0.473
Experiences with study procedures (maximum: 8)	7 (2–8)	3 (1–6)	3 (1–7)	<0.001
Experience vs expectations (maximum: 2)	1 (1–2)	1 (1–2)	1 (1–2)	0.778
Overall experiences (maximum: 7)	7 (6–7)	7 (6–7)	7 (6–7)	0.026
Total participant experience score (maximum: 60)	54 (49–57)	50 (44–54)	51 (46–56)	<0.001
Association between participant experience score and number of study visits attended				
Variable	Participant experience score			
	Correlation coefficient (*r*)		*P*-value	
Attendance of scheduled visits	0.20		<0.001	

### Satisfaction within themes

The highest thematic scores were satisfaction with study staff 6/6 (IQR 5–6) and satisfaction with study information 6/6 (IQR 4–6), Table 2. Conversely, satisfaction with study procedures yielded the lowest median score of 3/8 (IQR 1–7). This thematic score differed significantly between sites with Mbekweni yielding a lower median score [2/8 (IQR 0–4)] versus TC Newman [7/8 (IQR 3–8; *p* < 0.001)]. The intensive cohort also showed significantly lower satisfaction with study procedures, with a median score of 3/8 (IQR 1–6) compared to those in the main cohort [7/8 (IQR 2–8; *p* < 0.001)], Table 2.

### Reasons for joining the study

Reasons for joining the study were most commonly a belief that the study would improve the health of their child (99 %), that the study was important to friends’ or family’s health (97 %) or to receive better health care through participation (93 %), Table 3. Overall, 80 % of respondents indicated study reimbursements as “very important”; however, more Mbekweni mothers (88 %) cited this compared to TC Newman mothers (72 %, *p*-value <0.001). In addition, feeling pressured by others to join the study was significantly different between sites with 67 % of Mbekweni participants listing this as “very important” compared to 39 % at TC Newman, *p*-value <0.001.

**Table 3:** Reasons for joining and staying in the study

	Number (%) who responded that reason is “very important”			
Variable	Mbekweni	TC Newman	Total sample	*P*-value
*Reasons for joining the study*				
Significance of study topic to participant’s health or health of family/friends	289 (97)	275 (98)	564 (97)	0.842
Belief that participation will improve child’s health	296 (99)	281 (99)	577 (99)	1.000
For the financial incentive	259 (88)	204 (72)	463 (80)	<0.001
Receiving better health care services through participation	283 (94)	261 (93)	544 (93)	0.386
Prior positive experience participating in research	269 (91)	249 (88)	518 (89)	0.001
Hearing of others’ positive experience	260 (88)	224 (79)	484 (84)	0.009
Making a contribution to science	268 (91)	259 (92)	527 (91)	0.345
Feeling pressured by others to join	200 (67)	111 (39)	311 (54)	<0.001
Wanting to give back to the community	236 (84)	241 (89)	477 (87)	0.075
*Reasons for staying in the study*				
Close relationships with research staff	282 (95)	275 (96)	557 (96)	0.531
The study is interesting/the participant feels that she is learning	284 (96)	270 (96)	554 (96)	0.947
Feeling valued	276 (94)	268 (94)	544 (94)	0.696
Enjoying the individualized attention	268 (92)	264 (94)	532 (93)	0.847
Being treated better in research than in other settings	276 (93)	258 (91)	534 (92)	0.700
Having a chance to ask the research team about her/her child’s health	281 (95)	268 (95)	549 (95)	1.000
Learning about pregnancy and child development	285 (96)	269 (95)	554 (96)	0.432
Belief that her child’s health is better because of the study	289 (98)	277 (98)	566 (98)	0.905

### Reasons for staying in the study

Participant reasons ranked as “very important” for staying in the study were a belief that their child’s health was better for being in the study (98 %); close relationships with research staff (96 %); the opportunity to learn (96 %); and having the opportunity to discuss their or their child’s health with the research team (95 %), Table 3.

### Potential reasons for leaving

Common potential reasons for leaving the study were not receiving clinical or study results at study visits (31 %); inconvenience of attending study visits (27 %) and unanticipated aspects of the study (25 %), Table 4.

**Table 4:** Potential reasons for leaving

	Number (%) who responded that an experience was “difficult”			
Variable	Mbekweni	TC Newman	Total sample	*P*-value
Inconvenience of study visits	110 (37)	45 (16)	155 (27)	<0.001
Unanticipated aspects of the study	109 (36)	36 (13)	145 (25)	<0.001
Not receiving clinical test results	113 (38)	67 (24)	180 (31)	<0.001
Large number of study visits	78 (26)	24 (8)	102 (17)	<0.001
Long waiting time	104 (35)	21 (7)	125 (21)	<0.001
Lack of privacy	101 (34)	25 (9)	126 (22)	<0.001
Pressure to stay in the study	66 (22)	13 (5)	79 (14)	<0.001

### Factors influencing satisfaction

We investigated associations between participant characteristics and overall satisfaction scores. For unadjusted associations, participant characteristics significantly associated with higher overall satisfaction were recruitment from TC Newman clinic (β = 7.0; 95 % CI 6.0–8.1; *p* < 0.001), having completed secondary schooling (β = 3.1; 95%CI 1.8,4.3; *p* < 0.001), current employment (β = 2.3; 95 % CI 0.8,3.7; *p* = 0.002), higher SES (β = 5.3; 95 % CI 3.6,6.9; *p* < 0.001), and being above threshold for stressful life events (β = 3.0; 95 % CI 1.6,4.4; *p* < 0.001). Factors significantly associated with lower participant experience scores were prior participation in research (β = -6.1; 95 % CI -7.6, -4.6; *p* < 0.001) or enrolment in the intensive cohort (β = -4.6; 95 % CI -6.0,-3.2; *p* < 0.001). When fitting the multivariate model, clinic recruitment site was excluded as it was highly correlated with cohort. In the adjusted model, higher participant experience scores were associated with experiencing stressful life events in the past year (β = 2.3; 95 % CI 1.1,3.6; *p* < 0.001) and being in the highest SES category (β = 2.8; 95 % CI 0.7,4.8; *p* = 0.009). Factors significantly associated with lower satisfaction scores were prior research experience (β = -5.1; 95 % CI -6.6,-3.7; *p* < 0.001), and enrolment in the intensive cohort (β = -3.0; 95 % CI -4.3,-1.7; *p* < 0.001), Table 5. Also significant was the association between a higher satisfaction score and increased visit attendance (*r* = 0.20; *p* < 0.001), Table 2 (for the intensive cohort, only major study visit attendance was included, Fig. 1).

**Table 5:** Variables associated with higher participant experience score

		Unadjusted association			Adjusted association		
Variable	Median total score (IQR)	Regression coefficient	95 % CI	*P*-value	Regression coefficient	95 % CI	*P*-value
Clinic							
Mbekweni	47 (42–51)	Reference					
TC Newman	55 (50–58)	7.0	(6.0–8.1)	<0.001	-	-	-
Highest level of education							
Some secondary	50 (43–55)	Reference			Reference		
Completed secondary	52 (48–56)	3.1	(1.8–4.3)	<0.001	0.8	(-0.7-2.3)	0.269
Current employment status							
Unemployed	50 (45–55)	Reference			Reference		
Employed	52 (47–57)	2.3	(0.8–3.7)	0.002	0.7	(-0.8-2.1)	0.361
Composite SES quartile							
Lowest SES	48 (39–53)	Reference			Reference		
Low-moderate SES	50 (44–54)	2.5	(0.7–4.2)	0.005	1.4	(–0.2–3.0)	0.079
Moderate-high SES	51.5 (48–55)	3.9	(2.2–5.6)	<0.001	2.3	(0.6–4.0)	0.007
High SES	52 (48–58)	5.3	(3.6–6.9)	<0.001	2.8	(0.7–4.8)	0.009
Prior participation in research							
No prior participation in research	51 (47–56)	Reference			Reference		
Prior participation in research	45 (38–51)	-6.1	(-7.6 – -4.6)	<0.001	-5.1	(-6.6 – -3.7)	<0.001
Stressful events experienced							
Below threshold	50 (44–55)	Reference			Reference		
Above threshold^1^	53 (49–57)	3.0	(1.6–4.4)	<0.001	2.3	(1.1–3.6)	<0.001
Cohort							
Main cohort	54 (49–57)	Reference			Reference		
Intensive biweekly cohort	50 (44–54)	-4.6	(-6.0 – -3.2)	<0.001	-3.0	(-4.3 – -1.7)	<0.001

^1^Above threshold' defined as maternal report of havingexperienced 3 or more traumatic events

## Discussion

Understanding participant motivations for joining and staying in a research study is important to enrolment and retention, which are critical to conducting effective longitudinal research. Our findings include very high overall participant satisfaction, with the highest theme-specific scores being satisfaction with research staff and study information. Unsurprisingly, the lowest theme-specific satisfaction score was experience with study procedures. Participant experience with study procedures differed greatly by recruitment clinic and intensity of visit schedule. However, schedule intensity and recruitment clinic are highly correlated as almost all mothers at Mbekweni clinic attend bi-weekly visits compared to only half of mothers at TC Newman. Though there is evidence that the intensity of visit schedule affects participant satisfaction, the significant differences between clinics may also be the result of differences in information disseminated, differences in care given by staff or disparate cultural acceptance of the study and research procedures.

The negative aspects of study participation identified are common to research participation in general. Previous studies have found inconvenience, [10, 29] unanticipated aspects of participation [21] and not receiving test results to constitute barriers to participation [30]. Interestingly, participants were very satisfied with study information given but still had difficulty with unanticipated aspects. This may reflect the breadth of the study making it very difficult for staff to fully brief mothers on all aspects of participation. This also has implications for staff training on the consenting process, indicating the importance of consenting not only at pre-determined time points but as an on-going conversation. Study procedures including specimen collection (blood, swabs, induced sputum, tuberculosis skin tests) elicited negative responses in participants, as may be expected, given the potential discomfort to the child and time involved. Inconvenience is difficult to avoid as it is an inherent part of study participation. Operationally, this can and should be addressed to ensure that study schedules accommodate participants. In our context, a critical aspect of minimizing inconvenience is ensuring transportation assistance for participants. Aspects such as after-hours testing, weekend scheduling for working mothers or utilizing study drivers for participant transport are also potential approaches.

Unexpectedly prior participation in research was associated with lower satisfaction levels. Given the comprehensive nature and long duration of follow up within DCHS, those with prior research experience may have previously participated in research involving fewer follow up visits, fewer procedures and of shorter duration, and were therefore less satisfied with the high DCHS participant burden. However, the subset of participants with prior research experience (*n* = 105) was small and may be biased by another characteristic.

The association between lower satisfaction levels and intensive follow up likely reflects the significant burden of participation in the bi-weekly intensive cohort as well as discomfort associated with increased number of procedures done in the children in this group. Researchers should consider carefully follow up schedules and participant burden to appropriately weigh the benefit of additional data against inferior participant satisfaction. The relationship between higher satisfaction and high SES requires more investigation, but may reflect better understanding of the benefits of study participation to themselves and their community or improved scientific knowledge.

Critically, there was an association between improved attendance and higher participant experience scores. Though a limitation of this study is that the direction of causality cannot be determined, this significant association highlights the important relationship between participant satisfaction and improved visit attendance.

Participants’ high satisfaction with research staff likely reflects both close relationships with staff and perceived improvement in their child’s health. All mothers enrolled in the study receive primary care from public clinics that manage a large volume of patients, have very high staff to patient ratios and offer little opportunity to develop long standing relationships with patients. Previous studies have found that a significant motivator is trust in the study and study staff [31–33]. Our findings indicate that participants strongly trusted study staff and strongly perceived that study attendance enhanced their and their child’s healthcare.

The association between higher satisfaction and having experienced 3 or more stressful life events in the previous year may also relate to participant relationships with study staff, offering mothers a supportive environment that they might not have been able to access elsewhere. Literature suggests that research involving discussion of past trauma can be experienced as supportive and may be beneficial to participants [34]. Furthermore, mothers in this study were assessed for depression, past trauma and post traumatic stress disorder (PTSD). Where psychiatric symptoms where found, mothers were referred for follow up, fostering treatment in a population that may be under diagnosed or untreated. Study staff routinely referred participants for clinical treatment, including undiagnosed maternal illness and undiagnosed child illness (e.g. wheeze, pneumonia and tuberculosis), offering a first level screen and increased access to clinical staff for participants. Strong referral systems for psychiatric illness and childhood illness within DCHS as well as strong relationships with study staff may offer enhanced care compared to usual care, suggesting that the participant perception of personal benefit may be based on actual improvement in health.

Our data adds to a growing body of evidence that research participants, including healthy volunteers, are highly motivated by potential personal benefits, [29, 35, 36] even when no therapy is offered. Though there are discrepant data on whether altruism or perceived personal benefit is the stronger motivation for participant involvement in research, many studies have found a combination of the two to be an important motivator [14, 15, 17, 29, 35–37]. Researchers can capitalize on this by training staff to screen actively for illnesses, abnormal physical findings or harmful risk factors in their study population and by having strong referral systems, thus increasing the opportunity for direct personal benefit for participants. Linked to this is the importance of ensuring there are exist resources for treatment available to participants when physical illness or mental health issues are found.

Overall, maternal participant satisfaction was high. A limitation, however, is that evaluation of this experience was completed only after 16 months of involvement. This excludes those participants who were previously lost to follow up, who may have chosen to exit the study and who may have had worse experiences. However, there is a low loss to follow-up in the study, predominantly due to mothers moving out of the area and only a minority of participants indicated that multiple study aspects were difficult or uncomfortable. Ongoing evaluation of participant experiences may be useful as the study follow-up lengthens.

These findings suggest that healthy mothers in a LMIC have similar motivations for joining and staying in a research study compared to participants in RCTs and in high income countries [5, 7–9, 12–15]. Researchers should carefully consider follow up schedules and participant burden to improve satisfaction and consequently retention. Ongoing review of participant experience may be useful in informing study processes, especially given the correlation between improved attendance at follow up visits and higher satisfaction scores. Structured exit interviews would also add invaluable knowledge regarding why some participants choose to leave. Lastly, case referral and the opportunity for direct personal benefit as a motivator for participants remaining in research may be particularly relevant to longitudinal studies, which aim to keep participant engagement high over a long period of time.
